# A New Assay for Determining Ganglioside Sialyltransferase Activities Lactosylceramide-2,3-Sialyltransferase (SAT I) and Monosialylganglioside-2,3-Sialyltransferase (SAT IV)

**DOI:** 10.1371/journal.pone.0094206

**Published:** 2014-04-09

**Authors:** Cynthia Q. Sun, Ulrike Hubl, Petra Hoefakker, Madhusudan K. Vasudevamurthy, Keryn D. Johnson

**Affiliations:** Callaghan Innovation Research Ltd, Lower Hutt, New Zealand; University of Iowa, United States of America

## Abstract

A new assay for the determination of lactosylceramide-2,3-sialyltransferase (SAT I, EC 2.4.99.9) and monosialoganglioside sialyltransferase (SAT IV, EC 2.4.99.2) is described. The assay utilised the commercially available fluorophore labelled sphingolipids, boron dipyrromethene difluoride (BODIPY) lactosylceramide (LacCer), and BODIPY-monosialotetrahexosylganglioside (GM1) as the acceptor substrates, for SAT I and SAT IV, respectively. HPLC coupled with fluorescence detection was used to analyse product formation. The analysis was performed in a quick and automated fashion. The assay showed good linearity for both BODIPY sphingolipids with a quantitative detection limit of 0.05 pmol. The high sensitivity enabled the detection of SAT I and SAT IV activities as low as 0.001 μU, at least 200 fold lower than that of most radiometric assays. This new assay was applied to the screening of SAT I and SAT IV activities in ovine and bovine organs (liver, heart, kidney, and spleen). The results provided evidence that young animals, such as calves, start to produce ganglioside sialyltransferases as early as 7 days after parturition and that levels change during maturation. Among the organs tested from a bovine source, spleen had the highest specific ganglioside sialyltransferase activity. Due to the organ size, the greatest total ganglioside sialyltransferase activities (SAT I and SAT IV) were detected in the liver of both bovine and ovine origin.

## Introduction

Gangliosides are a class of complex glycosphingolipids that contain one or more sialic acid residues. They consist of a core ceramide structure and an oligosaccharide chain of varying length and complexity. These glycolipids are mostly located on the surface of the outer cell membrane of higher eukaryotic organisms from the echinoderms onwards. The level and structural composition of gangliosides is highly species and tissue specific and often changes during cell development and during various chronic disease states. They have been shown to be involved in many biological processes, including cell to cell recognition, transmembrane signaling, neuronal growth regulation and differentiation.[1], [2].

The biosynthesis of gangliosides is highly regulated and involves the successive action of highly specific glycosyltransferases including five sialyltransferases, SAT I, II, III, IV and V (Table S1). These enzymes are type II membrane proteins that are located in the membrane of the Golgi apparatus. They transfer sialic acid from the activated donor substrate cytidine 5'-monophospho-*N*-acetylneuraminic acid (CMP-NeuAc) to membrane-bound glycosphingolipid acceptors with high specificity [1], [3], [4]. The expression of these enzymes determines the level and the structure of the gangliosides produced in various cells and at different stages of development [5]. This is particularly apparent during brain development of young animals. At this stage, gangliosides are the major sialoglycoconjugates expressed in the central nervous system [6], [7]. Due to the increased demand, the biosynthesis of gangliosides is up-regulated including an increased expression level of sialyltransferases. Ganglioside GM3 is the precursor of more complex gangliosides and therefore plays a pivotal role in ganglioside synthesis. GM3 is solely synthesized by the action of the lactosylceramide-2,3-sialyltransferase SAT I (GM3 synthase). Low levels of SAT I activity leads to low levels of GM3 expression and therefore low levels of more complex gangliosides that are crucial for development. Recent research revealed that a deficiency of GM3 in humans resulted in infantile developmental stagnation and blindness [8]. Furthermore, the activity of ganglioside sialyltransferases, especially SAT I activity, is closely correlated with the cognitive skills and the development of memory and learning abilities of newborns [9], [10]. In adult life, studies have shown that gangliosides are important in the health of the neurological system [11], [12] as well as insulin signalling [13], [14]. However, over-expression of specific ganglioside sialyltransferases is associated with tumour formation and metastasis. The development of a highly sensitive specific sialyltransferase activity assay could enable detection of changes in the expression levels of these enzymes and therefore allow the early detection of diseases [15]–[17].

The traditional analysis method for ganglioside sialyltransferase activity involves the use of donor or acceptor molecules that are radiolabelled with ^14^C or ^3^H. This approach not only requires lengthy analytical procedures including TLC separation, solid phase extraction and scintillation photometry [18], [19] but also requires health and safety precautions when working with radioactive compounds. Alternatively, the use of fluoro- or chemo-labelled substrates (either CMP-NeuAc, or simple oligosaccharides) has been described [20]–[24]. However, these labelled substrates are generally not commercially available and require synthesis by the researcher. In addition, the use of labelled substrates representing the oligosaccharide acceptor for the detection of ganglioside sialyltransferases in complex biological samples has two major draw backs. Firstly, these methods are not specific for the activities of ganglioside sialyltransferases [20]–[24]. Sialyltransferases that are not involved in the ganglioside metabolism but share the same acceptor epitope (oligosaccharides only), would interfere with the results by reacting with the acceptor substrate. Secondly, ganglioside sialyltransferases usually engage the entire precursor ganglioside including the ceramide moiety for its enzymatic activity. In example, SAT I only reacts with lactosyl ceramide but not with lactose on its own clearly indicating the importance of the ceramide moiety. Therefore, the use of the labelled simple oligosaccharides as acceptor substrates might not give representative activity of the target enzyme. Complex samples containing endogenous substrates could also confound the results and reduce detection sensitivity.

In this paper, we present a highly sensitive method for determining ganglioside sialyltransferase activity in biological samples. The new assay uses commercially available sphingolipids (LacCer and GM1) labelled with boron dipyrromethene difluoride (BODIPY) as the acceptor substrates, which are specific for ganglioside sialyltransferase activity SAT I and monosialoganglioside-2,3-sialyltransferase (SAT IV), respectively). The high specificity of this assay made it possible to screen SAT I and SAT IV activity in biological samples with complex matrices. The utility of the assay is exemplified here by studying bovine and ovine organs, and will be widely applicable to evaluating activity in other systems.

## Materials and Methods

### Chemicals

The fluorophore labelled sphingolipids, BODIPY-GM1 and BODIPY-LacCer, with an excitation wavelength (λ_ex_) of 480 nm and an emission wavelength (λ_em_) of 510 nm, were from Invitrogen (Eugene, USA). The donor substrate, CMP-NeuAc, was of 99% purity and was supplied by Kyowa Hakko Kogyo Co. Ltd (Tokyo, Japan). All gangliosides (lactosylceramide, GM3, GD3, GM1 and GD1a) were from Matreya (PA, USA). 2-(*N*-morpholino)ethane-sulphonic acid (Mes) and MnCl_2_ were from BDH (Poole, England). β-Mercaptoethanol, Triton CF-54 and X-100 were from Sigma. These chemicals were of analytical grade. All the solvents used in the HPLC analysis were HPLC grade and from Scharlau (Barcelona, Spain).

Recombinant α2,3-(*O*)-sialyltransferase (EC 2.4.99.4) originating from rat liver was purchased from EMD Bioscience, as a reference for GD1a synthetase (SAT IV, EC 2.4.99.2) [25]. One unit is defined as the amount of enzyme that will transfer 1.0 μmol of sialic acid from CMP-NeuAc to Galβ1,3-GalNAcα1-benzyl per minute at 37°C in 2-(N-morpholino)ethane-sulfonic acid (Mes) buffer (25 mM, pH 6.0).

Bovine and ovine organs were collected fresh from a local abattoir (Taylor Preston, Wellington, New Zealand). Bovine samples taken from animals of an age of 7–30 days old were labelled as originating from calf. Animals older than a year were deemed to be adults. In the case of ovine samples, lambs were between 12 and 18 months old and adult sheep were around four years old.

### Sialyltransferase assays

Two separate sialyltransferase assays were developed in this study for the detection of SAT I and SAT IV using BODIPY-LacCer and BODIPY-GM1 as the acceptor substrates, respectively. CMP-NeuAc was the donor molecule in both assays. The reaction buffer for both assays contained 50 mM Mes in 0.1% Triton CF-54 or Triton X 100 (pH 6). BODIPY-LacCer and BODIPY-GM1 were transferred from the original vials to brown glass vials using ethanol. After removing ethanol under a N_2_ gas stream, the substrates were dissolved in the reaction buffer containing 144 μM CMP-NeuAc to give final concentrations of 13.75 μM and 31.58 μM for BODIPY-LacCer and BODIPY GM1, respectively. The mixtures were transferred as aliquots of 20 μl to small brown glass vials and stored at −20°C until required for the assay.

**Figure 1:**
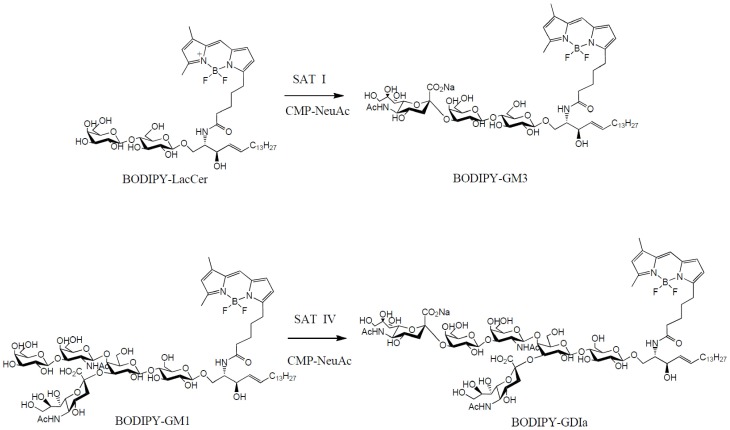
Enzymatic reaction schemes in the proposed ganglioside sialyltransferase assay; the reaction on the top describes the conversion of BODIPY-LacCer to BODIPY-GM3 by the action of SAT I; the reaction scheme below uses BODIPY-GM1 as the acceptor for determining the activity of SAT IV.

In a typical assay, 20 μl of reaction solution containing CMP-NeuAc and either BODIPY-LacCer or BODIPY-GM1 was incubated at 37°C. The reaction was initiated by the addition of 10 μl of enzyme solution. The incubation was carried out for 60 minutes at 37°C in a temperature controlled water bath (SW-21C, Julabo, Germany) under gentle shaking (200 rpm). The enzymatic reaction in this sialyltransferase assay is illustrated in Fig. 1.

After stopping the reaction by snap-freezing in liquid nitrogen, the frozen samples were dried using a concentrator (SpeedVac Plus, Savant SC210A, Thermo Scientific, USA). The glycolipids were extracted with 200 μl chloroform/methanol (1∶1, v/v). After centrifugation (10,000 rpm for 10 min, Centrifuge 5415D, Eppendorf) the supernatants were analyzed by HPLC as described below.

### HPLC analysis of BODIPY gangliosides and unlabelled gangliosides

The HPLC analysis was conducted on a Gilson system (Gilson, Middleton,Wisconsin, USA) with three pumps, a gradient mixer and an auto-sampler. The samples were separated using a YMC-Pac PVA-Sil column (250×4.6 mm, 5 μm, YMC, Japan) coupled with a guard cartridge (YMC-Pac Guard). Eluted BODIPY gangliosides were detected by a fluorescence detector (Shimadzu RF-10A XL) at λ_ex_ of 480 nm and a λ_em_ of 511 nm. An evaporative light scattering detector (PL-ELS1000, PolymerLab) was used for detecting unlabelled gangliosides. The HPLC operation and data processing were controlled by Gilson software (UniPoint 3.2).

The gangliosides were eluted at a flow rate of 1 ml/min using a gradient of solvents A (hexane/isopropanol, 98∶2 (v/v)), B (chloroform/isopropanol, 65∶35 (v/v) containing triethylamine (0.1%) and formic acid (0.027%)) and C (methanol containing triethylamine (0.1%) and formic acid (0.027%)). The detailed elution programs for LacCer and GM1 (BODIPY-labelled and unlabelled) are outlined in Table 1, respectively.

**Table 1:** HPLC gradient elution programs for the analysis of BODIPY gangliosides.

Table 1a	Analysis of BODIPY-LacCer and GM3			Table 1b	Analysis of BODIPY-GM1 and GD1a	
T (min)	A%	B%	C%	T (min)	B%	C%
0	60	40	0	0	80	20
3	60	40	0	3	80	20
5	0	100	0	34	20	80
25	0	40	60	35	0	100
30	0	0	100	39	0	100
33	0	0	100	40	80	20
34	0	100	0	41	80	20
35	0	100	0			
37	60	40	0			
39	60	40	0			

Eluents: A. hexane/isopropanol (98∶2); B. chloroform/isopropanol (65∶35); C. methanol.

B and C also contain triethylamine (1 mL/L) and formic acid (0.27 mL/L).

Flow rate: 1.0 mL/min. Injection volume: 20 μL.

Under the assumption that the addition of sialic acid to the BODIPY labelled substrate has no influence on the relative fluorescence intensity and caused no wavelength shift [26], the quantitative analysis of the peaks representing the reaction products was made using the standard curves obtained for the unreacted BODIPY substrates.

### Sialidase treatment of products from BODIPY assays

BODIPY-LacCer and BODIPY-GM1 were reacted with calf spleen microsomes (see below) and the reaction mixtures were separated by HPLC as described above. Peaks representing reaction products were collected, concentrated and analyzed by HPLC. The purity of the isolated products were 96.7% and 88.5% for the SAT I and SAT IV reaction, respectively. The purified products were dissolved in 50 μl distilled water. Then, 50 μl of sialidase solution (recombinant neuraminidase from *Clostridium perfringens*) at 1 U/μl in 50 mM citrate buffer at pH 4.5, an additional 50 μl of the same citrate buffer and 350 μl of distilled water were added. The mixtures were incubated for 18 hours at 37°C. The reactions were stopped by snap freezing in liquid nitrogen. After freeze-drying, the samples were dissolved in 100 μl chloroform/methanol (2∶1, v/v) and centrifuged. The supernatants were analyzed by HPLC. All measurements were performed at least in duplicate.

### Identification of BODIPY-labelled gangliosides by mass spectrometry (MS)

BODIPY-LacCer and BODIPY-GM1 (3.3 μmol) and CMP-NeuAc (7.5 μmol) in a total volume of 75 μl were incubated with microsome preparation at 37°C for 4 hours. The reaction mixtures were loaded on to Sep-Pak C18 cartridges (Phenomenex, USA). The cartridges were washed successively with distilled water (9 ml) and methanol (2 ml). Then the compounds were eluted with 2 ml of a mixture of chloroform/methanol (1∶1). The eluates obtained with methanol and chloroform/methanol were combined and the solvent was removed under a stream of N_2_. After extracting the majority of residual detergent (Triton) in the dried sample with dichloroethane (1 ml), the BODIPY gangliosides were recovered by adding 0.2 ml of chloroform/methanol (1∶2) [27].

The recovered BOIDPY gangliosides were separated on the HPLC as described above. Each peak representing BODIPY-LacCer, GM3, GM1 and GD1a were collected separately, then solvent removed and re-solubilised in 50 μl of methanol. Each fraction was analysed under both negative and positive mode by direct infusion into QTof MS. The MS analysis was operated on a Micromass QTof Premier Mass Spectrometer (Waters, MA, USA) equipped with an Electrospray ionization (ESI) and controlled by MassLynx software (version 4.1).

### Triton extraction of microsome fractions from bovine and ovine organs

The preparation of microsomes was based on the methods previously published in literature [28], [29] with modifications for the preservation of sialyltransferase activity. Details of the preparation are described in method S1.

Microsomes (1 g) were suspended in 15 ml of 25 mM Mes buffer (pH 6.2) containing 10% glycerol and 1% Triton CF-54 or Triton X-100 and sonicated twice on ice for 10 minutes with a 5 minute interval between treatments. The suspension was stirred for 2 hours at 4°C and then centrifuged at 52,000 *g* for 90 minutes at 4°C (Ti 70 rotor, Optima L-100XP, Beckman Coulter). The supernatant was collected and tested for ganglioside sialyltransferase activities.

### Determination of protein content

Bio-Rad DC protein assay kit (Hercules, CA, USA) was used for determining the protein concentration in samples containing detergents. The absorption was measured on a spectrophotometer (HEλIOSβ, Unicam) at a wavelength of 750 nm. Bovine serum albumin (BSA) from Bio-Rad was used as the protein standard.

## Results and Discussion

### Sialyltransferase assays using BODIPY labeled gangliosides as acceptors

In BODIPY-LacCer and BODIPY-GM1, the BODIPY fatty acid residue is linked to the ceramide chain (not the sphingosine chain) of the glycosphingolipid molecule (Fig. 1). This structural arrangement suggests free access of the sialyltransferases to the carbohydrate chain, while the ceramide group remains partially hydrophobic. BODIPY has good photostability with a fluorescence output at λ_ex_ 480 nm and λ_em_ 510 nm [30] The fluorometric HPLC analysis achieved good linearity (R^2^>0.99, Fig. S1) for both BODIPY glycosphingolipids with a limit of quantitation at 0.05 pmol [31], allowing the detection of SAT I and SAT IV activity as low as 0.001 μU using a one hour incubation period. The sensitivity of this method is comparable or even higher when compared to other fluorometric-based sialyltransferase assays (non-specific for SAT I and SAT IV activities) [20]–[24], and at least 200 fold higher when compared to the radiometric assays described for ganglioside sialyltransferases [18], [23].

The conditions for the separation of the products from the starting material by HPLC were optimised using unlabelled glycosphingolipids lactosylceramide (LacCer) and GM3 as well as GM1 and GD1a for SAT I and SAT IV reactions, respectively. The methods outlined in table 1 achieved good separation of the product peak from the acceptor substrate allowing accurate quantification of both peaks. When comparing the retention times for the unlabelled LacCer and GM1 with that obtained with the respective BODIPY-labelled compounds, no significant difference was observed indicating that the BODIPY group has little effect on retention times (Table 2). Therefore, the appearance of product peaks in the HPLC profiles with similar retention times to the unlabelled GM3 and GD1a indicated the formation of BODIPY-GM3 and BODIPY-GD1a respectively.

**Table 2:** Retention times (RT) of gangliosides and BODIPY gangliosides by two HPLC elution methods.

Gangliosides	RT (min) by elution program in Table 1a	RT (min) by elution program in Table 1b
LacCer	8.12	N.R.*
BODIPY-LacCer	8.18	N.R.
GM3	12.32	N.R.
BODIPY-GM3	12.39	N.R.
(identified peak)		
GM1	N.R.	13.96
BODIPY-GM1	N.R.	14.01
GD1a	N.R.	21.35
BODIPY-GD1a	N.R.	21.40
(identified peak)		

*not relevant.

**Figure 2:**
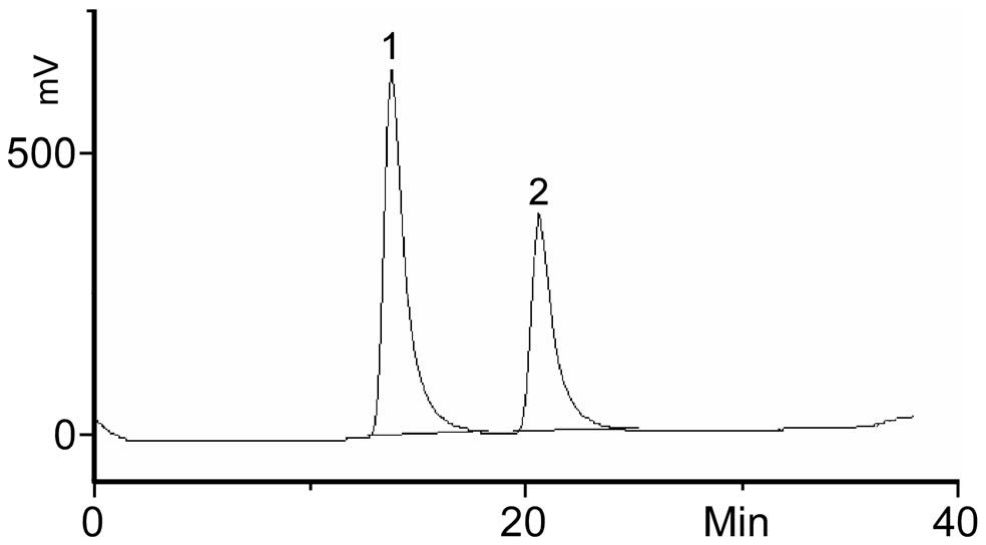
HPLC chromatogram of the mixture resulting from the reaction with rat liver 2,3-sialyltransferase; BODIPY-GM1 (76 μM) and CMP-NANA (144 μM) were incubated with rat liver 2,3-sialyltransferase (1.4 mU) for one hour at 37°C. The sample was snap-frozen, freeze-dried and dissolved in chloroform/methanol. The solution was analysed by HPLC. The product (peak 2) eluted after the acceptor substrate (peak 1) with baseline separation.

Purified enzymes are generally used for the determination of the optimal assay conditions, but neither SAT I nor SAT IV is commercially available. Pure rat liver α2,3-(O)-sialyltransferase (EC 2.4.99.4) was studied for assay optimization of SAT IV. This enzyme shares the Galβ1,3-GalNAc construct, which is identical to the terminal sugar construct in GM1 [25], [32], as the acceptor epitope with SAT IV and has been previously assumed to be identical to the latter. HPLC analysis of the products from the enzyme reaction showed the formation of a reaction product indicating that BODIPY-GM1 is a substrate for the rat liver enzyme (Fig. 2).

**Figure 3:**
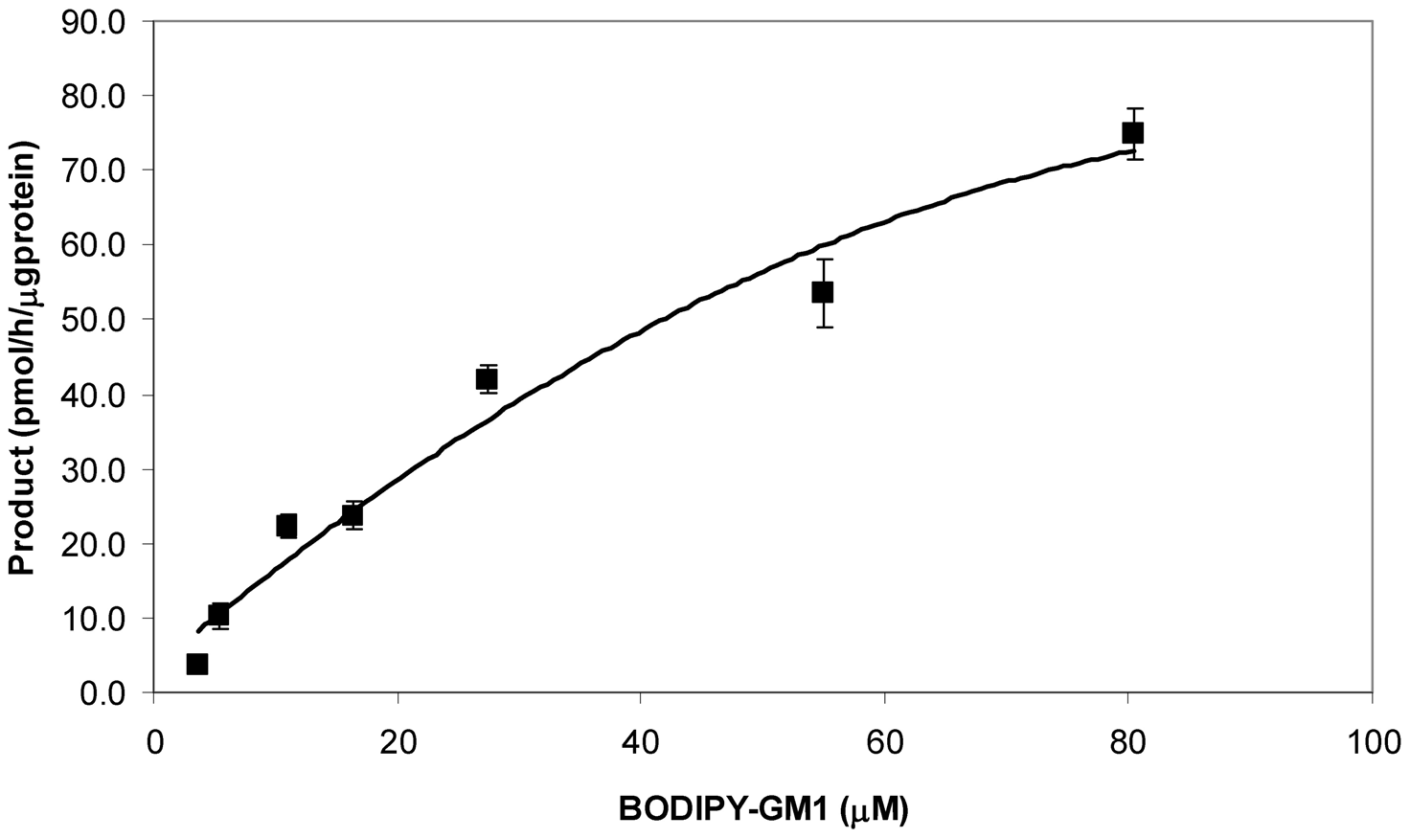
The effect of BODIPY-GM1 concentrations on the enzyme activity of rat liver 2,3-sialyltransferase; rat liver 2,3-sialyltransferase (1.4 mU) was incubated with a constant CMP-NeuAc concentration at 144 μM (1 h incubation, 37°C, pH 6) and varying concentrations of acceptor substrate BODIPY-GM1. Error bars indicate the differences between the high and low values of the duplicate samples.

**Figure 4:**
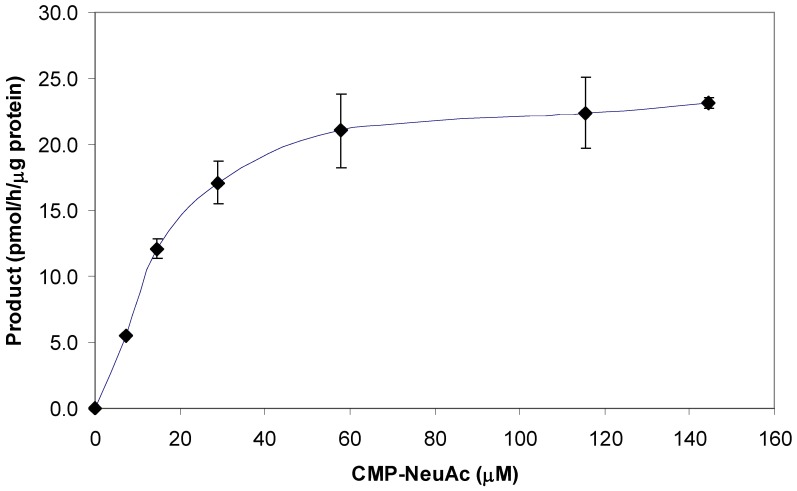
The effect of CMP-NANA concentration on the enzyme activity of rat liver 2,3-sialyltransferase; rat liver 2,3-sialyltransferase (1.4 mU) was incubated with a constant BODIPY-GM1 concentration of 13 μM (1 h incubation, 37°C, pH 6) and varying amounts of CMP-NeuAc. Error bars indicate the difference between the high and low values of the duplicate samples.

Different concentrations of BODIPY-GM1 and CMP-NeuAc, respectively, were incubated with 1.4 mU of the commercial α2,3-(O)-sialyltransferase for 1 hour. The conversion rates are shown in Figures 3 and 4. The Michaelis Menten constants were determined by using Lucenz version II. The *K*_m_ for BODIPY-GM1 was determined to be 97.9±0.4 μM, which was five fold lower than the *K*_m_ for unlabelled GM1 as determined by Lee and Kojima for similar sialyltransferases [33], [34]. The result for the *K*_m_ of CMP-NeuAc in this study was 23.4±0.1 μM, three fold lower than previously reported by Gu *et al* (65 μM) [35], however this was of a similar order of magnitude as data obtained for other sialyltransferases [24] This discrepancy most likely resulted from the use of different acceptor molecules in the individual studies. In addition, the enzyme in the previous study is a SAT IV whereas the rat liver enzyme in this study is an equivalent enzyme reacting primarily on O-glycans in glycoproteins.

Investigations on the mechanism of mammalian ganglioside sialyltransferases have indicated that the binding of the acceptor substrate not only involves the sugar moiety itself but also the ceramide residue. In studies on SAT I from rat liver and brain [36], [37] both fatty acid and sphingosine carbon chains of the LacCer ceramide backbone bound to the enzyme before NeuAc was transferred to the lactosyl group. The release of GM3 was rate limiting and dependent on the hydrophobicity of the carbon chains on the ceramide residue. The catalytic rate and release of GM3 was reduced with greater chain hydrophobicity. For rat liver SAT I, the *K*_m_ value for LacCer increased from 28 μM to 164 μM, and that for CMP-NeuAc increased from 77 μM to 857 μM with elongation of the fatty acid chain in the ceramide residue [36]. In the absence of pure SAT I enzymes of bovine and ovine origin, the kinetic data from the rat liver SAT I were considered as a reference for the selection of substrate concentrations used for SAT I assay development.

In comparison with unlabelled LacCer, BODIPY-LacCer is less hydrophobic because BODIPY partially replaced the fatty acid chain on the ceramide moiety. This would suggest that BODIPY-GM3 could be released faster than GM3 itself. Therefore, one could postulate that BODIPY-LacCer would be a better acceptor substrate than LacCer itself in the catalytic reaction with SAT I having a lower *K*_m_ value, as demonstrated in the case of BODIPY-GM1 for SAT IV in this study.

In the standard assay, concentrations of 13.75 μM and 31.58 μM were selected for BODIPY-LacCer and BODIPY-GM1, respectively. Although these values could be lower than the true *K*_m_ values of the BODIPY substrates for the SAT enzymes of different origin, the high sensitivity achieved in the fluorescent spectrometry based analysis enabled these concentrations for BODIPY-LacCer and -GM1 to be suitable for the screening in a complex biological matrix containing endogenous substrates.

The concentration for CMP-NeuAc in both SAT I and SAT IV standard assays was 144 μM, which is six times higher than its *K*_m_ value for the rat liver sialyltransferase (see above). This would ensure the saturation of the enzyme with the donor substrate and suggests that sialylation on the acceptor substrate would be rate limiting. It was found in this study that the enzyme inhibition from CMP-NeuAc occurred only when its concentration was greater than 280 μM.

### Identification of the products from the BODIPY sialyltransferase assay

The products of the reaction catalysed by SAT I and SAT IV are BODIPY-GM3 and BODIPY-GD1a, respectively. The identification of these products was challenging due to the lack of authentic standards for these compounds and the fact that only small quantities (nanogram) were formed in the standard enzymatic assay. As mentioned above, the incubation of the BODIPY-labelled glycosphingolipid substrates with the rat liver enzyme and the spleen microsomes yielded BODIPY-labelled product peaks which co-migrate with GD1a and GM3. Since the BODIPY-residue has little effect on the retention time of the respective glycosphingolipids this observation indicated the formation of the sialyltransferase product.

**Figure 5:**
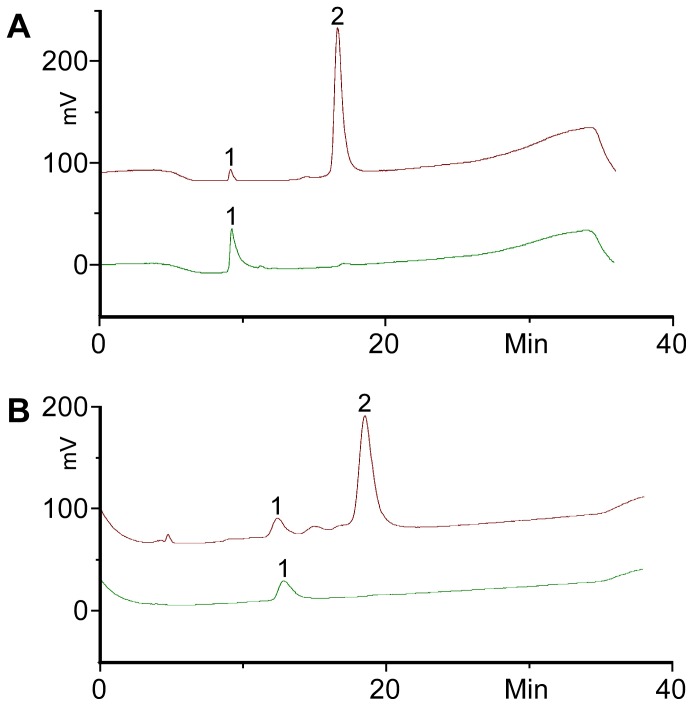
HPLC chromatograms of BODIPY-GM3 (A) and BODIPY-GD1a (B) recovered from enzyme reactions; reaction products from the sialyltransferase reactions were purified by HPLC and analysed (top trace) showing mainly the product (peak 2) and only little amounts of starting material (peak 1). The samples were treated with sialidase overnight at 37°C (lower trace, approximately 10-fold dilution from treatment before) and analysed showing complete conversion to the starting material (peak 1).

The identity of the products peaks was confirmed by treating the recovered products after HPLC with the sialidase from *C. perfringens*. This sialidase specifically hydrolyses α2,3 linked sialic acid. However, it does not react on the internal sialic acid in GD1a. HPLC analysis of the sialidase treated products, as shown in Figure 5A and 5B, revealed that the products were converted back to BODIPY-LacCer and BODIPY-GM1, respectively, based on their HPLC retention times. This result showed that the products from the SAT I and SAT IV assays contained one additional 2,3-linked sialic acid when compared to the starting materials BODIPY-LacCer and BODIPY-GM1, and the products were BODIPY-GM3 and BODIPY-GD1a, respectively.

**Figure 6:**
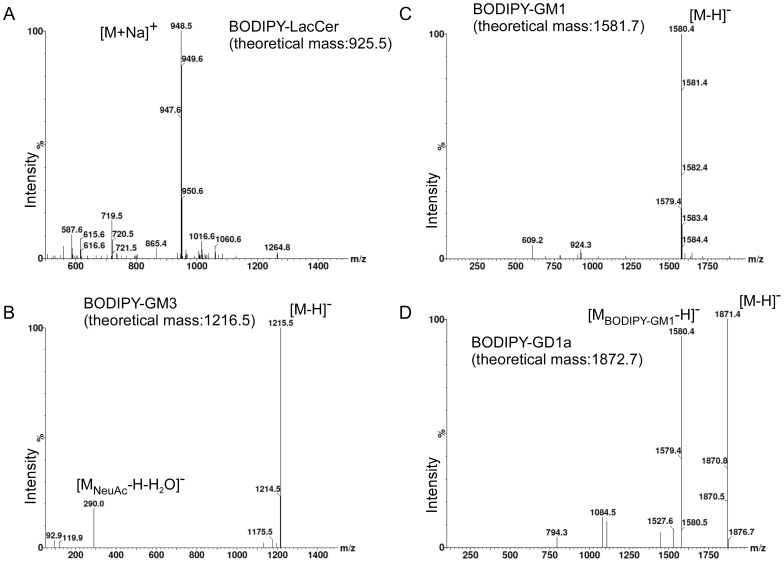
QTof MS and MS/MS analysis of the BODIPY labelled substrates and products in SAT I and SAT IV catalysed reactions. (A) MS spectra of BODIPY-LacCer in positive mode; (B) MS/MS spectra of BODIPY-GM3 in negative mode; (C) MS spectra of BODIPY-GM1 in negative mode; (D) MS/MS spectra of BODIPY-GD1a in negative mode.

In addition, the collected HPLC fractions of the SAT assay products were analysed by MS. The samples contained comparatively high concentrations of Triton X100 (0.1–1%) in contrast to low concentration of the BODIPY-glycoshingolipids (nanogram) making the detection and identification of product related signals in the MS difficult. Therefore,the detergent was removed according to Suzuki and Kabayama [27]. MS analysis on the collected HPLC fractions showed signals for masses corresponding to BODIPY-LacCer, BODIPY-GM3, BODIPY-GM1 and BODIPY-GD1a, respectively (Fig. 6). The structures of the peaks corresponding to BODIPY-GM3 and BODIPY-GD1a were further confirmed by the fragmentation pattern of the respective molecular ions in the MSMS analysis. In the case of BODIPY-GM3, the presence of the fragmentation ion of NeuAc (m/z 290) indicates the sialylation of the substrate BODIPY-LacCer. The fragmentation of the molecular ion representing BODIPY-GD1a (m/z^-^1871) revealed a major peak at m/z 1580 which corresponds to the acceptor substrate (BODIPY-GM1) and has been generated by the loss of NeuAc.

### Detection of SAT I and SAT IV activities in bovine and ovine organs

Four types of organs (liver, spleen, heart and kidney) from bovine and ovine sources were processed, resulting in 11 microsome preparations. The details of the yields of microsomes (g/wet weight) and protein levels are summarised in table S2. The TLC analysis of the microsome samples obtained in bovine and ovine organs showed the presence of a variety of glycosphingolipids including LacCer, GM3 and GD1a (method S2 and Fig. S2). This indicated that endogenous substrates may interfere with the sialyltransferase assay.

Sialyltransferases were solubilised from the microsome preparations by sonication and stirring in the presence of surfactants (Triton CF-54 or Triton X-100) as described in Materials and Methods [33]–[35], [38].

**Figure 7:**
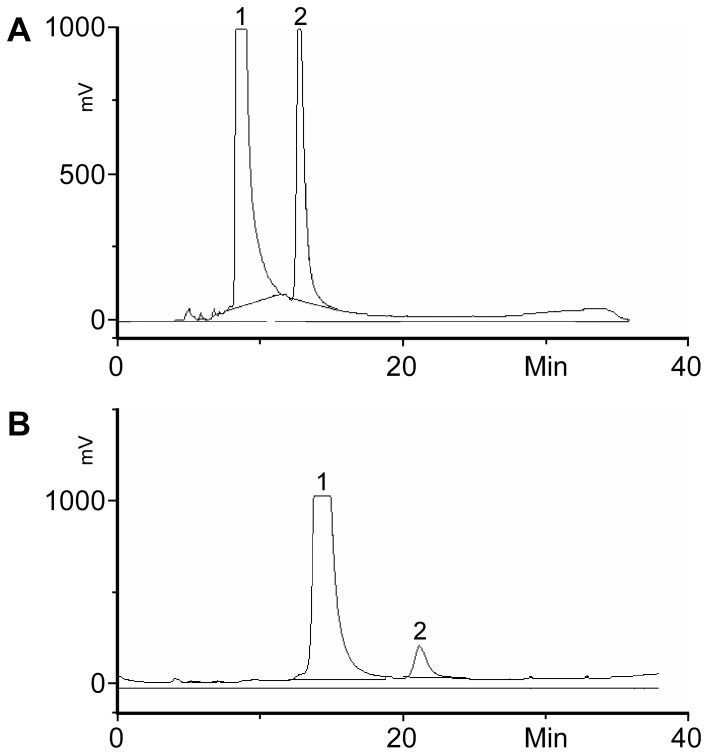
HPLC chromatograms of SAT I (A) and SAT IV (B) assays with calf spleen microsomes; calf spleen microsomes were extracted with Triton. The resulting extract was tested for SAT I (A) and SAT IV (B) activity by incubation with the respective BODIPY-labelled substrates for 1 h at 37°C. The presence of the respective activity was indicated by the presence of the product (peak 2) next to unconverted substrate (peak 1).

Detection of SAT I and SAT IV activity was conducted separately. The reactions were carried out at 37°C for 1 hour and overnight (18 hours). Fig. 7 (A & B) shows the chromatograms after a 1 h reaction with a Triton extract from a calf spleen microsome preparation. In both assays, an additional peak appeared after incubation, which eluted after the substrate. The new peaks were identified as BODIPY-GM3 and BODIPY-GD1a, respectively, via their retention times, sialidase treatment and MS analysis (see above), suggesting that both SAT I and SAT IV activities were present in the Triton extracted samples.

The results from the activity screening of SAT I and SAT IV in bovine and ovine organs samples are summarized in Table 3. Due to the extremely low level of enzyme activities found in heart, the results obtained from overnight experiments are also included for reference. However, the specific enzyme activities obtained from 1 hour reaction were more representative. The data demonstrate that the assay can distinguish a diverse range of activity levels found in different organs. Although the investigation was preliminary in nature, the results indicated that young animals, such as calves, produce sialyltransferases as early as seven days after parturition with the highest specific activity located in the spleen for both enzymes. After maturation (>12 months), the production of ganglioside sialyltransferases increased in the liver, while a significant drop of enzyme activity (about 2 fold) was observed in spleen. However, the specific activities of both SAT I and SAT IV in spleen are still approximately twofold higher when compared to the same activities in bovine liver.

**Table 3:** Activities of SAT I and SAT IV in Triton extracted fractions from different bovine and ovine organ microsomes sourced from the raw material preparation in Table S2.

Organ source	SAT I activity (1 h)*	SAT I activity (18 h)*	SAT IV activity (1 h)*	SAT IV activity (18 h)*	Total activity (18 h) of SAT I per organ	Total activity (18 h) of SAT IV per organ
Bovine liver	0.06±0.01	0.09±0.02	0.06±0.01	0.12±0.04	12607	16109
Bovine kidney	0.04±0.01	0.06±0.01	0.03±0.01	0.14±0.01	559	2497
Bovine spleen	0.11±0.02	0.23±0.05	0.09±0.01	0.27±0.09	2127	2497
Bovine heart	n.d^a^	0.01±0.00	0.02±0.00	0.27±0.02	6.0	160
Calf spleen	0.25±0.02	0.37±0.06	0.24±0.02	0.67±0.05	853	1566
Calf liver	n.d^a^	0.05±0.02	n.d^a^	0.02±0.01	830	369
Ovine liver	0.12±0.01	0.22±0.03	0.06±0.01	0.15±0.01	6243	4399
Ovine spleen	0.06±0.01	0.17±0.02	n.t^b^	n.t^b^	1269	n.a^c^
Ovine kidney	0.02±0.01	0.04±0.01	0.05±0.01	0.09±0.01	58	130
Ovine heart	n.d^a^	0.01±0.00	0.02±0.01	0.18±0.02	5.4	97
Lamb spleen	0.07±0.00	0.15±0.00	n.t^b^	n.t^b^	682	n.a^c^

Activity is defined as 1 nmol of product formed per mg of protein in the defined period of time.

*The data were an average from two measurements. The error represents the differences between the high and low values of the duplicate samples.

a not detected; ^b^not tested; ^c^data not available.

Similar levels of SAT I and IV activities were observed in lamb and sheep spleens. This may be due to fact that the lambs tested were more than 12 months old and changes regarding SAT I and SAT IV production due to maturation may have already occurred. The hearts from both bovine and ovine sources had the lowest activity for either SAT I and SAT IV. If the size of the organ tested was taken into consideration, bovine and ovine liver were considered to be the best source for total SAT I and SAT IV activities.

These findings have not been reported in the literature. Further studies on a larger sample pool, more defined age groups, as well as other organs including brain, would be required to fully understand the profiles of ganglioside sialyltransferases during the development of young animals.

## Conclusions

In mammals, the biosynthesis of gangliosides is highly regulated, which is reflected in the expression levels of highly specific sialyltransferases. These enzymes have specific structural requirements regarding the glycosphingolipid acceptor substrate, including not only the carbohydrate chain but also the ceramide residue. Therefore, an assay using acceptor substrates containing not just the carbohydrate chain but the entire structural elements of the ganglioside molecule is often needed for accurate assessment of the activity of a particular ganglioside sialyltransferase enzyme.

The novel assay developed in this study utilized BODIPY labelled sphingolipids as acceptor substrates for determining SAT I and SAT IV enzyme activities. BODIPY-labelled sphingolipids have been widely used as probes in the study of lipid trafficking in living cells [26], [39] and have been proven to be stable in the environment of a complex bio-matrix. When the assay was conducted overnight, no degradation of starting material (BODIPY-LacCer or BODIPY-GM1) was observed. The use of BODIPY labelled acceptor molecules allows the distinction between different sialyltransferase activities. The lower *K*_m_ value of BODIPY-GM1 in comparison to unlabeled substrate as demonstrated in this study, and the considerations regarding the structures of BODIPY-LacCer and LacCer, suggest that BODIPY-LacCer and BODIPY-GM1 could be competitive acceptors for SAT I and SAT IV, respectively. The potential interference from endogenous substrates in complex biological samples could thus be minimized. The application of this assay in the detection of SAT I and SAT IV activities in bovine and ovine organs demonstrated its high selectivity and sensitivity in complex biological samples. The low amounts of substrates (at pmol level) needed in this assay will be of benefit during the purification procedure of SAT I and SAT IV from biological sources, as well as to the performance of kinetic studies.

In summary, a new assay was developed suitable for fast screening of specific ganglioside sialyltransferase activities (SAT I and SAT IV) in crude biological matrices which contain gangliosides as potential endogenous substrates. The high sensitivity of this assay could potentially enable it to be applied in the diagnosis of diseases that are associated with altered ganglioside sialyltransferase activity, such as neurological disorders and tumour formation.

## Supporting Information

Figure S1 **Standard curves of BODIPY-LacCer (▪) and BODIPY-GM1 (▴).** The relative standard deviations for the data shown on the graphs were smaller than 3.9% (n = 3).
(TIF)

Figure S2 **The TLC analysis of bovine microsomes Triton extracted samples.** Lane 1: ganglioside standards (GM1, GD3 & GD1a); Lanes 2 & 3, calf liver microsomes Triton extract; Lane 4: LacCer standard; Lanes 5–8: calf spleen microsomes Triton extract; Lane 9: Standards of LacCer and GM3.
(TIF)

Table S1 **Sialyltransferases involved in the biosynthetic pathway of gangliosides.**
(DOC)

Table S2 **preparation of microsomes from different bovine and ovine organs.**
(DOC)

Method S1 **Procedure for the preparation of microsomes from bovine and ovine organs.**
(DOC)

Method S2 **TLC analysis of glycosphingolipids in microsomes from bovine and ovine organs.**
(DOC)
