# Supplementary figures and images for: A New Assay for Determining Ganglioside Sialyltransferase Activities Lactosylceramide-2,3-Sialyltransferase (SAT I) and Monosialylganglioside-2,3-Sialyltransferase (SAT IV)
Source: PLoS One. 2014 Apr 9;9(4):e94206. doi: 10.1371/journal.pone.0094206 (PMC3981761; doi:10.1371/journal.pone.0094206)

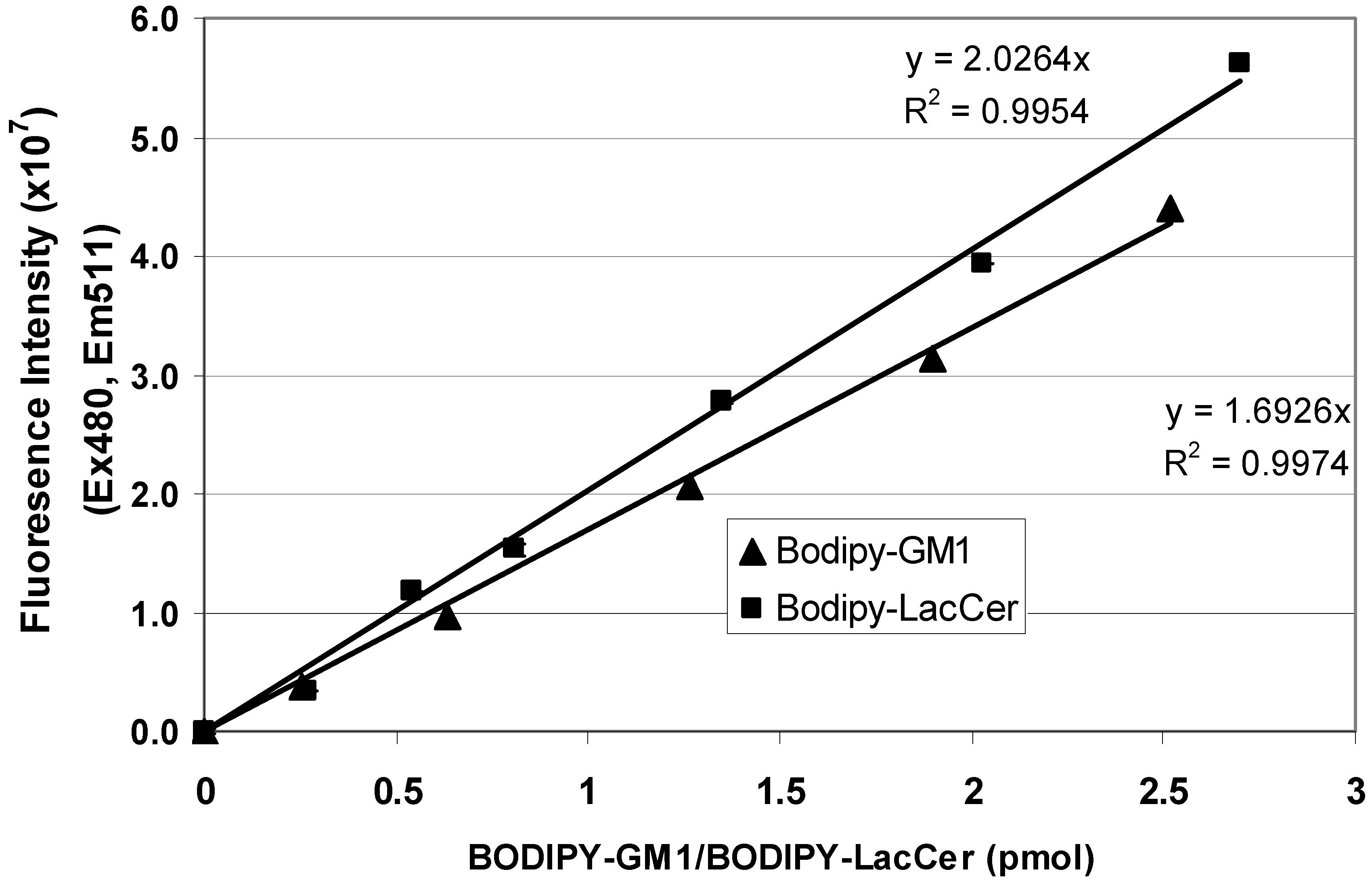

Supplement: Figure S1 — Standard curves of BODIPY-LacCer (▪) and BODIPY-GM1 (▴). The relative standard deviations for the data shown on the graphs were smaller than 3.9% (n = 3). (TIF) [file pone.0094206.s001.tif]
