# Supplementary material for: A New Assay for Determining Ganglioside Sialyltransferase Activities Lactosylceramide-2,3-Sialyltransferase (SAT I) and Monosialylganglioside-2,3-Sialyltransferase (SAT IV)
Source: PLoS One. 2014 Apr 9;9(4):e94206. doi: 10.1371/journal.pone.0094206 (PMC3981761; doi:10.1371/journal.pone.0094206)
Supplement: Table S1 — Sialyltransferases involved in the biosynthetic pathway of gangliosides. (DOC) [file pone.0094206.s003.doc]

**SUPPLEMENTARY DATA Table S1.**

Ganglioside sialyltransferases (summarized from NC-IUBMB website: <http://www.chem.qmul.ac.uk/iubmb/enzyme/>)

| **Enzyme Name** | **EC No.** | **Acceptor** | **Comments*** |
| --- | --- | --- | --- |
| lactosylceramide- α 2,3-sialyltransferase | 2.4.99.9 | Gal-Glc-Cer | SAT I, GM3 synthase. Lactose itself cannot act as acceptor. |
| Sial-α 2,8-sialyltransferase | 2.4.99.8 | Sial-2,3-Gal-Glc-Cer | SATII, GD3 synthase. Maybe SAT III as well. |
| monosialoganglioside sialyltransferase | 2.4.99.2 | Gal-GalNAc-Sialyl-Gal-Glc-Cer | SATIV, GD1a synthase |
| α 2,8-sialyltransferase | 2.4.99.8 | Sialα2,3-Galβ1,3-GalNAcβ1,4-(Sialα2,3-)Galβ1,4-Glcβ1–Cer | SAT V, GT1a synthase, transfer of sialic acid to a terminal sialic acid. |

*SAT I, II, III, IV, and V are the five sialyltransferases involved in the ganglioside synthesis pathway. CMP-sialic acid is the donor substrate for all sialyltransferases.
