# Supplementary material for: A New Assay for Determining Ganglioside Sialyltransferase Activities Lactosylceramide-2,3-Sialyltransferase (SAT I) and Monosialylganglioside-2,3-Sialyltransferase (SAT IV)
Source: PLoS One. 2014 Apr 9;9(4):e94206. doi: 10.1371/journal.pone.0094206 (PMC3981761; doi:10.1371/journal.pone.0094206)
Supplement: Table S2 — preparation of microsomes from different bovine and ovine organs. (DOC) [file pone.0094206.s004.doc]

**SUPPLEMENTARY DATA Table S2**

Preparation of microsomes from various animal organs.

| Animal (age) | Organ | Raw material (g) | Microsome obtained (g) (%)^b^ | Protein in microsomes (mg/g) ^c^ |
| --- | --- | --- | --- | --- |
| Calf | Liver | 769.3 | 33.1 (4.2%) | 558 |
| (7-30 days) | Spleen (4)^a^ | 411.3 | 15.7 (3.8%) | 597 |
| Cattle | Liver | 4471.5 | 238.5 (5.3%) | 587 |
| (12-18 months) | Kidney | 420.1 | 18.2 (4.3%) | 560 |
|  | Spleen (2)^a^ | 992.1 | 27.6 (2.7%) | 669 |
|  | Heart | 900.6 | 2.4 (0.3%) | 251 |
| Sheep | Liver | 625.3 | 37.1 (5.9%) | 765 |
| (4 years) | Spleen | 425.0 | 8.9 (2.1%) | 864 |
|  | Kidney (12)^a^ | 844.8 | 28.1 (3.3%) | 621 |
|  | Heart | 632.4 | 2.3 (0.4%) | 234 |
| Lamb  (12-18 months) | Spleen | 370.0 | 8.3 (2.2%) | 548 |

^a^: The number of organs if not one piece, used in one preparation.

^b^: Microsome yield (percentage % in starting raw material).

^c^: Data for protein concentrations has a relative standard deviation (RSD) <3% (n=3).
