# Supplementary material for: A New Assay for Determining Ganglioside Sialyltransferase Activities Lactosylceramide-2,3-Sialyltransferase (SAT I) and Monosialylganglioside-2,3-Sialyltransferase (SAT IV)
Source: PLoS One. 2014 Apr 9;9(4):e94206. doi: 10.1371/journal.pone.0094206 (PMC3981761; doi:10.1371/journal.pone.0094206)
Supplement: Method S1 — Procedure for the preparation of microsomes from bovine and ovine organs. (DOC) [file pone.0094206.s005.doc]

**SUPPLEMENTARY DATA METHOD S1**

***Preparation of microsomes from ovine and bovine organs***

The preparation of microsomes was based on the methods previously published in literature [26,27] with modifications for the preservation of sialyltransferase activity.All preparation steps were carried out at 4 °C. Briefly, fresh organs (~1 kg) were cut up and the white fibrous material was removed. The wet weight was determined and the organ pieces were then homogenized in a chilled sucrose solution (0.25 M) with three bursts of 45 seconds each at the highest setting of a Waring blender. The homogenate was filtered through a coarse sieve into a beaker and centrifuged for 30 min at 5000 *g* (Sorvall RC5, GS3 rotor). The supernatant was decanted into a large beaker and a solution containing MnCl_2_ (50 mM) was added at a ratio of 10% (v/v) of the total supernatant volume. After stirring the supernatant for 20 minutes, the precipitated microsomes were collected by centrifugation (9000 *g*, 90 min). The microsome pellets were suspended in Mes buffer (25 mM buffer, pH 6.7) containing 0.2 mM β-mercaptoethanol. The pellet was thoroughly dispersed using a spatula and vigorous stirring to ensure homogeneity. Additional buffer was added until the homogenate was smooth. Finally, the homogenate was freeze-dried. The dry microsomes were collected and stored at –80 ºC until required.
