# Supplementary material for: A New Assay for Determining Ganglioside Sialyltransferase Activities Lactosylceramide-2,3-Sialyltransferase (SAT I) and Monosialylganglioside-2,3-Sialyltransferase (SAT IV)
Source: PLoS One. 2014 Apr 9;9(4):e94206. doi: 10.1371/journal.pone.0094206 (PMC3981761; doi:10.1371/journal.pone.0094206)
Supplement: Method S2 — TLC analysis of glycosphingolipids in microsomes from bovine and ovine organs. (DOC) [file pone.0094206.s006.doc]

**SUPPLEMENTARY DATA METHOD S2**

***Gangliosides analysis by TLC***

TLC analysis of unlabelled gangliosides was performed on HPTLC plates (Nano-Sil 20, 10x10 cm, Macherey-Nagel, Düren, Germany) using a mixture of chloroform, methanol, and aqueous 0.2% CaCl_2_ (55:45:10, v/v/v). After drying, the gangliosides were visualized using a copper spray (10% CuSO_4_ in 8% aqueous H_3_PO_4_) and heating at 170 °C for 10 minutes. The stained plates were photographed using an imaging system from Alpha Innotech (DE-500 cabinet with a camera) under UV light (360 nm) and using a filter at 520 nm. The density of the bands representing the gangliosides was quantified by Alphaimager software 1220 V5.5.
